# Exploring the impact of optical corrections on visual functions in myopia control–a scoping review

**DOI:** 10.1007/s10792-024-02937-w

**Published:** 2024-02-09

**Authors:** Salai Dhavamathi Janarthanan, Kaleem Samiyullah, Gopinath Madheswaran, Shonraj Ballae Ganeshrao, Kathleen Watt

**Affiliations:** 1 Department of Optometry, Manipal College of Health Professions, Manipal Academy of Higher Education, Manipal, Karnataka India; 2 School of Optometry and Vision Science, Faculty of Medicine and Health, Univeristy of New South Wales, Sydney, Australia; 3 Acchutha Eye Care & Institute of Optometry, Erode, Tamil Nadu India

**Keywords:** Peripheral defocus spectacles, Orthokeratology lenses, Multifocal contact lens, Contrast sensitivity function, High-contrast visual acuity, Low contrast visual acuity, Scoping review

## Abstract

**Purpose:**

Myopia is controlled optically with peripheral defocus spectacles, multifocal contact lenses, or orthokeratology lenses. However, it is unknown which optical correction will improve visual performance. This scoping review aimed to identify and summarize studies on various visual functions using optical corrections for myopia control.

**Methods:**

To develop the search strategy, population (Myopia), concept (visual performance), and context (unrestricted race/region) were used. PubMed, SCOPUS, Cochrane Library, and Web of Science databases were searched using the keywords myopia, contrast sensitivity, high and low contrast visual acuity, stereopsis, and optical correction of myopia control. This scoping review protocol was registered in the Open Science Framework registry and followed the framework for scoping review outlined by the Joanna Briggs Institute.

**Results:**

Eight studies (*n* = 8) met the inclusion criteria and were included in the review. Four were conducted in Europe, two were conducted in China, and one was conducted in Japan and Singapore. Five studies were randomized controlled trials, out of which three used contact lenses and two studies used peripheral defocus spectacles lenses. Studies ranged from one day to 2 years. Three studies that used orthokeratology lenses were prospective study designs. Among the studies that used orthokeratology lenses and contact lenses, two studies measured the contrast sensitivity function with CSV1000 (Vector Vision) under mesopic and photopic conditions, with and without glare. Two studies measured the central and peripheral contrast sensitivity using psychophysics experiments. High and low contrast visual acuity was measured using the Freiburg Vision Test (*n* = 1) and ETDRS charts (*n* = 3), and stereopsis was assessed using a random dot stereogram (*n* = 1). The studies showed a reduction in central and peripheral contrast sensitivity function and low contrast acuity when treated with multifocal contact lenses, orthokeratology lenses, and peripheral defocus lenses compared with single-vision lenses.

**Conclusion:**

This scoping review found a reduction in central and peripheral contrast sensitivity function, as well as low contrast visual acuity when using various optical corrections for myopia control, while high-contrast visual acuity remained the same. The impact of visual functions may not influence the effectiveness of myopia control. Eye care practitioners should provide awareness to the parent and patient population about the potential visual impact of recent designs for optical corrections of myopia control.

**Supplementary Information:**

The online version contains supplementary material available at 10.1007/s10792-024-02937-w.

## Introduction

Myopia is a rising worldwide issue, highly prevalent in the cities of East and Southeast Asia [1, 2] Even though the prevalence of myopia has increased significantly in East Asian regions, the myopia epidemic in India has gone unnoticed. Myopia prevalence grew dramatically among urban children (aged 5 to 15 years) from 4.5% in 1999 to 21.2% in 2019. According to recent reports, myopia increased by at least 0.25D in approximately 50% of Indian children each year and by approximately a dioptre in 18%. Myopia has been estimated to continue to increase in prevalence, reaching 31.89% in 2030, 40.01% in 2040, and 48.14% in 2050 [2].The most common progressive eye disorder tends to occur in younger individuals. There is a rise in the ratio of high myopia, which is related to an increased risk of ocular problems such as cataracts, glaucoma, macular degeneration, retinal detachment, and choroidal neovascularization.

Myopia can be classified based on the severity and age of onset. The myopia severity is further classified as low (−0.50D to −3.00D), moderate myopia (− 3.00D and −6.00D), and high myopia (more than − 6.00 D) [3, 4].

Myopia is categorized depending on the age of onset as juvenile, young, or school myopia, which develops all through the time of adolescence to early young years (8–14 years) [5, 6].

Interventions to avoid or postpone the commencement of myopia, and/or to slow its progression are critical to minimize the adverse visual effects of complicated ocular pathologies. Even though myopia progression management methods are rising in number, proof of treatment efficacy is variable. Myopia progression can be conventionally slowed or halted through optical, pharmacological, environmental, and low-level light therapy treatments.

Myopia decreases visual performance due to optical correction forms and retinal changes. Optical correction for myopia includes the latest technology spectacle lenses, multifocal contact lenses, and orthokeratology lenses. High-contrast acuity is commonly used to assess visual performance; however, other functions are also important to measure in a clinical setting. Functions include stereopsis, contrast sensitivity function, and low contrast acuity.

Eye care professionals may often find it challenging to recommend the best optical treatment for myopic patients. However, there is limited evidence available to determine the treatment methodology that offers the best visual performance.

Addressing this query can be challenging in clinical practice, especially when myopic patients inquire about the treatment modality that provides the best vision. In most clinical settings, high-contrast visual acuity is the commonly assessed measurement. While many patients may have good high-contrast visual acuity (VA), they often complain of blurred vision. Visual acuity measures the ability to perceive small details, which remains a crucial indicator of visual function in clinical evaluation and research. However, contrast sensitivity has been demonstrated to be a more accurate measure of visual performance than visual acuity, particularly in tasks related to daily living and recognizing the visual environment [7].

High myopic subjects have been observed to have noticeably impaired contrast sensitivity function [7, 8]. A comprehensive assessment of a person's vision-related abilities requires the study and characteristics of both visual function and functional vision [9, 10]. Many latest technology optical corrections are available in clinical settings to slow the progression of myopia such as Defocus Incorporated Multiple Segment Lenses (DIMS), orthokeratology lenses, multifocal contact lens, and bifocal or multifocal spectacles. Several tests have been performed and are being processed to determine the effectiveness of these lenses. It is critical to understand how these lenses affect visual performances. As a result, the visual function examinations will aid in gaining a better grasp of the subject's level of vision quality when wearing these lenses.

The primary goals of this scoping review are as follows: (a) to comprehend the impact of optical myopic progression treatments on visual performance including contrast sensitivity, high and low contrast visual acuity, and stereopsis; (b) to determine whether the visual performance can aid clinical decision making in myopia control treatments; (c) to map the research done in this field systematically; and (d) to identify gaps in the existing literature.

## Methodology

The scoping review protocol was registered in the Open Science Framework registry (OSF) [11] and adhered to the Joanna Briggs Institute (JBI) scoping review framework [12]. The authors have done the following processes: [1] identify the research objectives; [2] identify relevant studies; [3] screen and choose studies; [4] chart the data; and [5] collate, summarize, and report the results.

### Identification of the research objective

The study objectives mentioned in the introduction have been formalized. The population, concept, and context (PCC) method created a search strategy.

- Population: People with myopia.
- Concept: Visual performance.
- Context: There are no restrictions on gender, ethnicity, or geographic area.

### Identification of relevant studies

Electronic records were searched using, Cochrane Library, PubMed, Web of Science, and SCOPUS. The database search terms were “myopia,” “visual functions,” “optical interventions,” contrast sensitivity functions and “high and low contrast acuity.” Other sources, such as thesis, dissertations, and gray literature, were also searched. There were unrestricted ethnicity or geographic regions.

### Screening and selection of studies

The titles and abstracts of the identified papers were screened using the inclusion and exclusion criteria listed below.

This review included subjects with myopia who were defined and classified by Filtcroft et al. [13]. Myopia was optically controlled with peripheral defocus spectacles, multifocal contact lenses, or orthokeratology lenses, studies that measured the impact of contrast sensitivity, high and low contrast acuity, stereopsis, and visual performance using optically controlled myopic lenses were included. The review excluded subjects with other retinal pathologies, studies that did not define myopia, did not measure visual functions, and did not provide a clear concept and methodology. Those studies which were not published in full text in scientific journals in English and in conference abstracts. Duplicates were removed by importing the studies into Mendeley Desktop [14]. Two writers exported the studies to Microsoft Excel 2013 [15] for administration and selection based on titles and abstracts. Studies were independently selected and were not influenced by the decisions of other authors. Studies that did not meet the criteria were removed. When a decision on inclusion or exclusion was different between the two reviewers, a third reviewer was consulted and reached a consensus and resolved these process differences. The full text of the targeted studies was obtained and read. In addition, citations from the extracted papers were searched using inclusion and exclusion criteria. The study selection process was documented in a flowchart according to the Preferred Items Guidelines for Systematic Review and Meta-Analysis Reports for Scoping Reviews (PRISMA-ScR) [16].

### Charting the data

The data from the selected studies were mapped using a given form using Microsoft Excel 2013. Data extracted from the selected studies included study demographics (author, year of publication), methodology (purpose, sample size, and population), outcomes, and main results. Data extraction and mapping were performed independently by two authors and reviewed by a third author.

Two authors categorized and collated the important information from the included research, which was then validated and accepted by a third author. Finally, existing research needs in visual functions to know the impact of visual performance with the peripheral defocus spectacle lenses, multifocal contact lenses, and orthokeratology lenses.

## Results

**Fig. 1:**
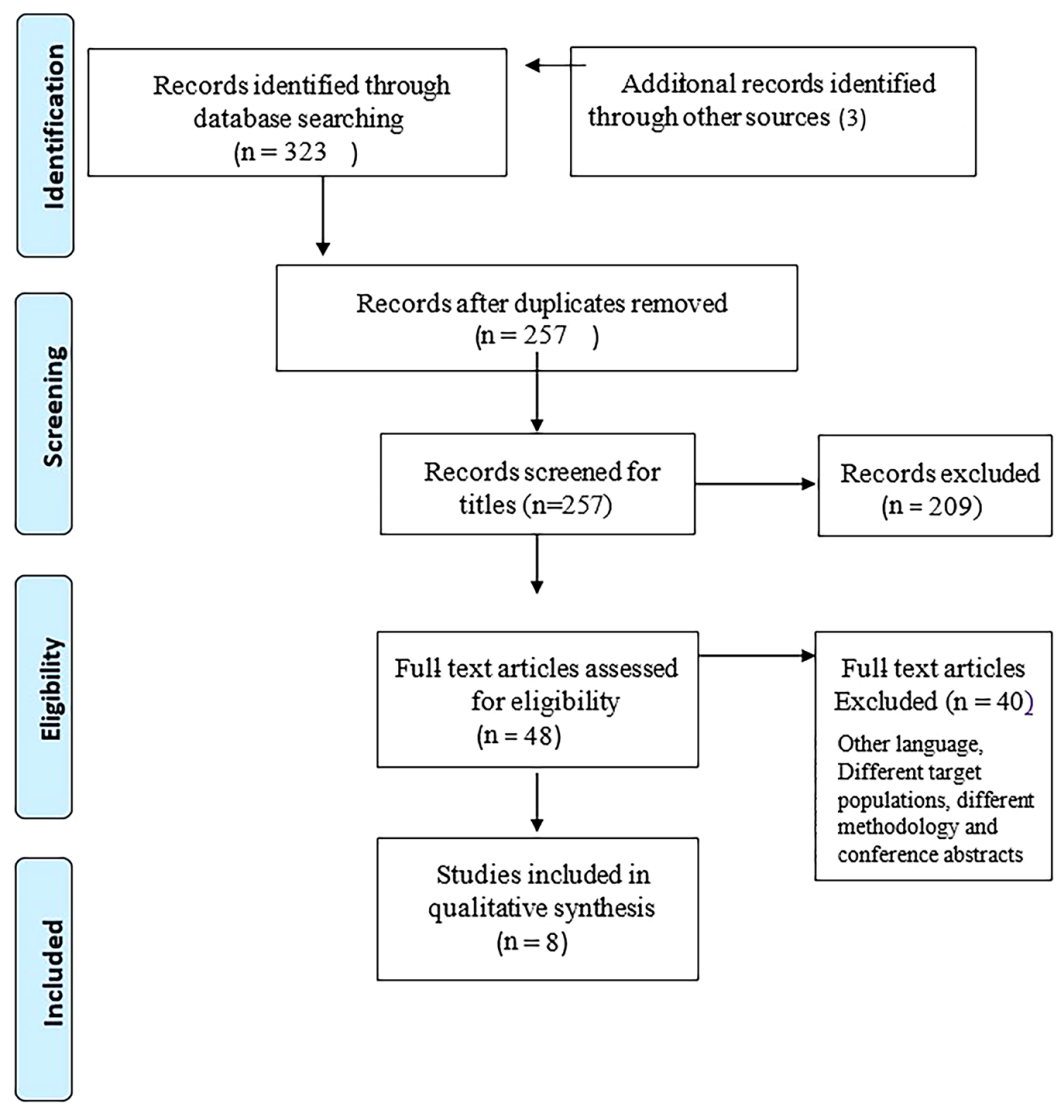
Flowchart on literature search and study selection

The authors retrieved 332 titles and abstracts from four different electronic databases. Additionally, three articles were found from the references of the retrieved articles. Mendeley citation manager [17] was used for abstract and title screening, removing duplicates, and citations. After removing 66 duplicate records, 257 titles were reviewed for eligibility and 209 were excluded. Full-text articles 48 were screened for eligibility. A total of 40 articles were excluded. The major reasons for exclusion were procedural and methodological differences, conference abstracts, diverse targets, and reviews. Finally, eight articles were included for the qualitative synthesis as the primary outcome was a visual function in myopia control treatment strategies (Fig. 1).

Table 1 represents the nature of the studies designated, which provided the visual functions in subjects with myopia control optical treatment strategies. Out of these eight studies, four studies were conducted in the East Asian population and four studies from the European population. Most of the studies were directed at subjects between the age (8–37 years) compared to the younger and older population. The visual functions were available in all eight articles, and a few other tests were also carried out, like MTF (Modulation Transfer Function) Accommodative Lag, and Motion Perception.

**Table 1:** Shows the characteristics of eight reviewed articles

Study details	Age (years)	Participants (number of eyes)	Location of report
Kobayashi et al. [20]	21–37	30	Japan
Ehseai et al. [21]	20–32	36	Finland
Guo et al. [22]	8–14	44	Helsinki
Liu et al. [23]	9–11	27	China
Przekoracka et al. [24]	18–36	48	Poland
Lam et al. [18]	8–13	320	China
Gao et al. [19]	19–47	56	Singapore
Nti et al. [25]	21–29	50	Spain

These review results were obtained after compiling the results from the published literature, which had measured the visual functions in various myopia control optical treatment strategies. The demographic details such as age, sample size (N), and ethnicity of each variable are given in detail Table 1

Table 2 describes the various visual function parameters measured instruments used for measuring the parameters. The table also shows the type of optical aid used for myopia control and the refractive error range. The mean refractive error was found to be −4.37D.

**Table 2:** Shows the parameters measured, instruments, optical aid used and degree of myopia. SVCL (Single-Vision Contact lenses) HAL (Highly Aspherical Lenslets), SAL (Slightly Aspherical Lenslets),

S no	Study details	Parameters measured	Instruments used	Optical aid used	Refractive error range (Spherical equivalent)
1	Kobayashi et al. [20]	Contrast sensitivity under photopic conditions	CSV1000 (Vector Vision)	Orthokeratology lenses	−1.00 D to −4.00D
2	Ehseai et al. [21]	Visual acuity and central and peripheral visual performance	p-scan 100 system (Psychophysical methods)	Daily disposable single-vision contact lenses and single-vision spectacle lenses	Mean (−4.88D to −8.75D)
3	Guo et al. [22]	Contrast sensitivity under scotopic and mesopic condition, Aberrations and MTF	Nidek opd-scan iii, Vector vision, CSV-1000e	Orthokeratology lenses	− 1.25 D to − 4.25 D
4	Liu et al. [23]	Corneal topography, CSF, MTF, Strehl ratio (sr), Objective Scattering Index	Double-pass optical quality analysis system, Pentacam, CSV-1000e	Orthokeratology lenses	−0.75 D to −5.50 D
5	Przekoracka et al. [24]	Visual Acuity at Distance and Near and Central and Peripheral Contrast Sensitivity at Distance	Landolt C Optotypes and Gabor patch tests	Multifocal soft contact lens (RELAX, Swiss and SVCL)	− 0.25 D to − 6.75 D
6	Lam et al. [18]	High and low contrast visual acuity and Accommodation Lag and Stereopsis	Early treatment diabetic retinopathy logarithmic 2000 series charts, Howell phoria distance and near card, RAF ruler	DIMS lens and Single-Vision Spectacles	−1.00 D to −5.00D
7	Gao et al. [19]	High and Low contrast visual acuity and Motion perception	LogMar and Gabor patch test	HAL, SAL, single-vision lens	−8.50 D to + 1.75D
8	Nti et al. [25]	Distance and near contrast sensitivity under photopic and mesopic conditions	Psychophysical methods	Biofinity single-vision contact lens (SVCL), Biofinity D Multifocal + 2.50 add, and NaturalVue Multifocal	−0.75 D to −6.00 D

Table 3 depicts the visual functions like low and high-contrast visual acuity, contrast sensitivity function under mesopic and scotopic conditions, as primary outcomes which were measured in each of the optical interventions used for myopia control.

**Table 3:** Shows the difference among visual functions in each of the optical interventions used for myopia control

Outcomes	Kobayashi et al. [20]	Ehseai et al. [21]	Guo et al. [22]	Liu et al. [23]	Przekoracka et al. [24]	Lam et al. [18]	Gao et al. [19]	Nti et al. [25]
Optical intervention used	Orthokeratology	Single-vision spectacles & contact lenses	Orthokeratology	Orthokeratology	Multifocal contact lenses	DIMS	HAL,SAL	Multifocal soft contact lenses
High contrast Visual acuity distance	↑	=	↑		↓	=	=	
Visual acuity near					=	=	↓	
Low contrast visual acuity		=					↓	
Contrast sensitivity scotopic			↓		↓	=		↓
Contrast sensitivity mesopic	↓		↑	↓	↑	=		=

= : No difference noted, ↓: Significant decrease in the experimental group. ↑: Significant increase in the experimental group

## Discussion

This scoping review summarizes the important studies on the impact of visual functions using the myopia control lenses in the results section. The discussion has been categorized as follows: Visual functions with novel designs myopia control spectacle lenses and contact lenses.

The key aspects tested in visual functions in all of these tests were contrast sensitivity function, high and low contrast visual acuity, corneal curvature, stereopsis, and amplitude of accommodation. Several types of optical correction have been employed in studies to control myopia progression. With the defocus incorporated multiple segment lenses (DIMS), visual contrast sensitivity (VCS), showed no decrease in VCS compared with a single-vision lens, in both photopic and mesopic conditions [18]. A study that evaluated visual contrast sensitivity with three different lenses showed a smaller impact on VCS with HAL (Highly Aspheric Lenslet) and SAL (Slightly Aspheric Lenslet) lenses than with DIMS lenses [19].

This review included parameters such as multifocal contact lenses, daily disposable contact lenses, spectacle lenses, orthokeratology lenses, single-vision contact lenses, HAL and SAL, and DIMS spectacle lenses. Subjects' refractive errors ranged from -0.25D to -6.00 dioptres depending on the spherical equivalent of astigmatism. Landolt C optotypes, Gabor patch test, psychophysical methods used P-scan 100 system and Aston contrast sensitivity test, Nidek OPD- scan, CSV-1000e, Pentacam, and ETDRS charts were used to examine individuals' objective and subjective responses. The population tested range in age from 8 to 37 years and were  from Chinese, Japanese, Spanish, Polish, Finnish, and Singaporean origin. Out of all the visual functions examined, most studies demonstrate that using myopia control optical aids does not enhance all visual functions. Few studies suggest some degree of visual functions were affected. The main thing to consider here is that either most studies have assessed the short-term effects of the aids or the sample size is very small. Few of the visual functions are subjective measurements from the patients, minimal diversity, only mild-to-moderate myopes have been assessed, no clarity among the types of myopia, and no proper comparison between the established lens and new lens considering all of this, the compilation of all research concludes that using latest technology lenses designed for myopia progression showed a reduction in the visual function which is not statistically significant of the patients exposed to it.

## Strengths and limitations

Scoping review methodology allowed for the collection of diverse literature with a comprehensive search using five databases. However, the review also had several limitations. First, the subjective nature of the article selection due to the scoping review methodology to collect all evidence that might contribute to the study aim. Second, only studies that used the latest technology designs for optical treatment for myopia control were considered; all the studies are done on a different cohort of subjects including some outside the range of patients usually treated with myopia progression control treatments. Finally, abstracts from conferences were excluded; however, many abstracts were later published in full text.

Despite these limitations, this scoping review followed the rigorous methodology advocated by JBI guidelines. This study recommends comparing the visual functions of various optical modalities of myopia control treatment on the same cohort of subjects for further considerations.

## Summary

The present study conducted a comprehensive scoping review on the impact of myopia control optical interventions on visual functions. The review process involved a systematic search and screening of relevant literature from multiple electronic databases. Eight articles were included for qualitative synthesis after thorough screening and exclusion of ineligible studies.

The included studies were diverse in terms of population ethnicity and age range. Three studies were conducted in East Asian populations, while others included subjects from various European countries. The age range of participants varied between 8 and 37 years, with one study extending beyond 40 years. Visual functions were the primary outcome in all eight articles, and additional tests such as modulation transfer function (MTF), accommodative lag, and motion perception were also conducted in some studies.

The various visual function parameters measured, the instruments used for these measurements, and the type of optical aid employed for myopia control were presented in the results. Contrast sensitivity function and low- and high-contrast visual acuity were the most commonly measured parameters across the studies. Other measurements included visual acuity at distance and near, central and peripheral contrast sensitivity, stereopsis, motion perception, corneal topography, and aberrations. The mean refractive error across the studies was approximately -4.37D.

Different optical aids showed varying effects on visual functions. Some interventions were found to enhance visual functions, while others had a slight impact. However, it was noted that even with interventions that impacted visual functions, there was an increase in visual acuity, indicating potential benefits for myopia control.

The impact of spectacle interventions on visual functions varied, with some interventions resulting in no significant difference, while others showed a slight decrease or increase in specific visual functions.

## Conclusions

Overall, the findings from the scoping review suggest that myopia control optical interventions can have different effects on visual functions. While some aids had no significant impact, others had a minor effect while improving visual clarity. It is important to note that most of the studies included in the review had evaluated short-term effects or were limited by small sample sizes. Additionally, the ethnic diversity of the populations studied was limited, and the range of myopia severity mainly was mild to moderate.

Despite these limitations, the review provides valuable insights into the impact of myopia control optical interventions on visual functions. The findings may assist eye care professionals in making informed decisions when prescribing these interventions for myopia control. However, further research with larger and diverse populations and long-term follow-ups is needed to fully understand the effects of these interventions on visual functions and their efficacy in controlling myopia progression. The study concludes that the impact of visual functions may not influence the effectiveness of myopia control. Eye care practitioners should provide awareness to the parent and patient population about the potential visual impact of recent designs for optical corrections of myopia control.

## Supplementary Information

Below is the link to the electronic supplementary material.

Supplementary file1 (DOCX 17 KB)

## Data Availability

Only published articles were acquired for this scoping review. Data supporting this review is publicly available.

## Code availability

NA.
