# Supplementary material for: Exploring the impact of optical corrections on visual functions in myopia control–a scoping review
Source: Int Ophthalmol. 2024 Feb 9;44(1):47. doi: 10.1007/s10792-024-02937-w (PMC10858094; doi:10.1007/s10792-024-02937-w)
Supplement: Supplementary file 1 — Supplementary file1 (DOCX 17 KB) [file 10792_2024_2937_MOESM1_ESM.docx]

**Supplementary file :1 Database with search strategy-** Last search on : 21st February 2023

### PubMed :No of hits:84

(((((visual acuity) AND (visual function)) AND (contrast sensitivity)) AND (myopia control lens)) OR (orthokeratology)) OR (multifocal lens)

**Cochrane Library**:**No of hits hits:34**

("visual acuity"):ti,ab,kw AND (visual function):ti,ab,kw OR (contrast sensitvity):ti,ab,kw AND (myopia control lens):ti,ab,kw AND (orthokeratology lens)

**Web of science :No of hits :12**

high contrast visual acuity (All Fields) AND contrast sensitivity (All Fields) AND myopia control lens (All Fields) AND visual function (All Fields)

**Scopus :No of hits :184**

( TITLE-ABS-KEY ( visual  AND  acuity )  AND  TITLE-ABS-KEY ( contrast  AND  sensitivity )  OR  TITLE-ABS-KEY ( myopia  AND  control  AND  lens )  OR  TITLE-ABS-KEY ( orthokeratology  AND  lens )  OR  TITLE-ABS-KEY ( multifocal  AND  lens )  AND  TITLE-ABS-KEY ( visual  AND  function )  AND  TITLE-ABS-KEY ( high  AND contrast  AND visual  AND acuity ) )

**Other reference :No of hits :18**

visual acuity and contrast sensitivity and myopia control lens and orthokeratology and dims lens
